# The Effect of Shared Decision‐Making on Emergency Management Knowledge, Anxiety, and Mental Health Among Family Members of Terminally Ill Patients in the ICU: A Quasiexperimental Study

**DOI:** 10.1155/nrp/8910437

**Published:** 2026-03-10

**Authors:** Hui-Ying Cheng, Shu Yuan Chao, Hsin-Hung Chen

**Affiliations:** ^1^ Department of Nursing, Zuoying Armed Forces General Hospital, Kaohsiung, 813204, Taiwan; ^2^ Department of Nursing, Hungkuang University, Taichung, 43302, Taiwan, hk.edu.tw; ^3^ Department of Medical Education and Research, Kaohsiung Veterans General Hospital, Kaohsiung, 813414, Taiwan, vghks.gov.tw

**Keywords:** anxiety, emergency management, family members, ICU, knowledge, mental health, shared decision-making

## Abstract

Shared decision‐making (SDM) in intensive care units (ICUs) aids family decision‐making and mental health; its impact on emergency management knowledge, anxiety, and mental health is unclear. In a quasi‐experimental pre–post study at a teaching hospital in southern Taiwan, 60 family members of terminally ill ICU patients (30 SDM, 30 control) were enrolled. The SDM group received a three‐talk model intervention (choice, options, decision talk); the control group received usual care. Emergency management knowledge, anxiety, and mental health were assessed via self‐administered questionnaires before and after the intervention. Data were analyzed using Mann–Whitney U and Wilcoxon signed‐rank tests and multivariable linear regression. In the SDM group, emergency management knowledge increased from a pretest mean of 16.87 (SD 3.45) to a post‐test mean of 19.33 (SD 1.49), albeit statistically significant (*p* < 0.05). Anxiety scores rose in the SDM group (post‐test mean 47.13, SD 4.77) versus a slight decrease in controls (post‐test mean 43.63, SD 6.56), with no significant intergroup difference (*p* = 0.284). Mental health scores (a secondary outcome) declined in both groups, indicating persistent emotional distress. After adjusting for confounders, the SDM intervention remained a significant predictor of increased knowledge. SDM enhanced knowledge of emergency management but did not alleviate anxiety or improve mental health among ICU family members. Integrating targeted emotional support into SDM models warrants exploration.

## 1. Introduction

The intensive care units (ICUs) [1, 2] are special hospital wards that provide care to critically ill patients, with the major goal of maintaining the life and health of patients, aggressive pain control, and improving the quality of life as the second goal [3]. Despite the rapid advancement of medical technology, life‐sustaining therapies in ICU may not be able to meet patients’ goals. Because most of their relatives do not understand the real situation of the disease condition nor have enough knowledge for decision‐making, they will be shocked and unable to accept the sudden deterioration of the disease or even the death of their loved ones. Insufficient communication may also lead to misunderstandings and conflicts among family members and result in a situation in which patients receive various emergency measures [4]. Previous studies have shown that ICU deaths account for 20% of patient deaths in U.S. hospitals, and the concept of dying with dignity has emerged in recent years [5]. In Taiwan, the overall ICU mortality rate is approximately 20.2%, highlighting the importance of quality end‐of‐life care in this setting [6].

The primary goal of end‐of‐life care is to improve life quality, not prolong life indefinitely [7]. An important quality‐of‐life indicator in end‐of‐life care includes living with caregivers, pain intensity, physical activity, spirituality, and social support [8]. Indicators of palliative care include symptom control, place of death, proactivity in care, end‐of‐life decisions, and healthcare cost [9]. It is not an easy task to initiate end‐of‐life planning. Because the Chinese generally consider it bad luck to talk about death, it is a challenge for clinical staff to talk about end‐of‐life planning with the patients. Terminally ill patients may lose their decision‐making ability and not be able to participate in the decision of medical treatments [10]. There has been globally an increasing emphasis on helping terminally ill patients die with dignity and minimizing the family members’ regrets of losing their loved ones. When caring for terminally ill patients, family members suffer various emotional stresses, such as anxiety, depression, and post‐traumatic stress disorder (PTSD) [11]. Interdisciplinary cooperation is key to supporting seriously ill patients and families and to smooth communication with the families, such that the healthcare team would be able to help and encourage them and address their concerns about the patient’s disease condition, decision‐making, and alternative solutions [12].

“Shared decision‐making” (SDM) is a patient‐centered approach where healthcare providers and the patient work together to make the best healthcare decision for the patient [13]. This is a frequent strategy for diseases that are highly uncertain, complicated, or with multiple treatment options, and it provides the most complete information for patients or family members, reduces the family members’ anxiety, and provides humanized care for terminally ill patients [14]. During a patient’s stay in the ICU, ICU practitioners should activate preventive measures as early as possible, provide complete information, communicate with the patient’s families, and improve the quality of intensive care. “Shared decision‐making” is still in the initial stage, and studies focusing on the implementation and achievements are limited [15]. As senior clinical practitioners, we fully understand what the family members encounter when making decisions. Therefore, this study aimed to evaluate the effectiveness of an SDM intervention on family members’ knowledge of emergency management, anxiety, and mental health.

## 2. Methods

This pre–post‐intervention study, reported in accordance with the TREND (Transparent Reporting of Evaluations with Nonrandomized Designs) statement [16], compares the effectiveness of SDM with a traditional intervention on families’ knowledge of emergency management, anxiety, and mental health in ICU patients.

### 2.1. Intervention Measures

The experimental group carried out “SDM.” In this study, SDM is adapted from the three‐talk model proposed by Elwyn et al., which includes team talk, option talk, and decision talk [17, 18]. The research team comprises interdisciplinary medical staff, i.e., physicians, nurses, palliative care nurses, social workers, nutritionists, and respiratory therapists. The attending physician in the ICU assessed the patients as terminally ill and matched the criteria for the SDM study. The researchers introduced themselves to the patient’s family members, and then the research assistants explained the study’s aim and the family members’ rights. After they had signed the informed consent form, the researchers presented the SDM procedure to the participants. The first part, which included the team talk and option talk, lasted approximately 30–40 min and covered the introduction of the disease, treatment options, and the pros and cons of each option. In the following discussion, participants are free to ask questions for clarification and to gain a deeper understanding of the options. After the explanation and discussion, the participants (family members) brought the SDM sheets home. The second part, decision talk, was scheduled for the day following the first meeting, lasted approximately 20–30 min, and was conducted by the same research team members to ensure consistency. In the control group, traditional care intervention was implemented, i.e., the medical group provided explanations to the patient’s family members when they asked questions about emergency management.

### 2.2. Subjects

The population was derived from terminally ill patients in a research hospital in Southern Taiwan. A quasi‐experimental, nonequivalent control group design was used. Participants were assigned to groups based on the calendar month of ICU admission. Family members of patients admitted in even‐numbered months were assigned to the experimental group, while those admitted in odd‐numbered months were assigned to the control group. The recruitment process involved first identifying patients in the ICU who were terminally sick and met the study criteria and then approaching their family members for participation. Terminally ill patients were defined as those with a progressive, irreversible illness who, in the clinical judgment of the attending ICU physician, had a life expectancy of less than 6 months and for whom curative treatment was no longer considered a viable option. Patient disease severity was assessed using the Acute Physiology and Chronic Health Evaluation II (APACHE II) score upon ICU admission. It was coded and targeted to experimental and control groups that matched the inclusion criteria and were recruited without signing the DNR or predocumented orders. The inclusion criteria are above 20 years old, first‐ to third‐degree relatives of the patient, agree to participate in this study, have clear consciousness, can communicate in Mandarin Chinese or Taiwanese, and have the ability to answer questions correctly. The sample size was calculated using G Power software, with an effect size of *f* = 0.25 (a medium effect size according to Cohen’s guidelines [19], appropriate for exploratory research without prior local data), a power level of 0.80, and a significance level of 0.05. Anticipating potential attrition due to patient death and family distress in the ICU setting, we initially recruited 78 participants to ensure adequate final sample size. During the study, 11 patients died and 7 family members declined to complete the post‐test, resulting in 60 valid samples (30 per group). Therefore, 30 cases were collected for each group in this study from January to December 2022. Thirty minutes before the visit, participants were invited to the ICU consultation room in a teaching hospital in Southern Taiwan, where researchers explained SDM intervention to the participants and their families. While the homogeneity of the groups was supported by demographic data, we acknowledge that a more detailed assessment of the family support network was not performed and represents a study limitation.

### 2.3. Research Tools

This study collected data via self‐administered questionnaires consisting of basic information, emergency management, anxiety, and mental health scales. The basic information scale comprises gender, age, level of education, religious belief, occupation, etc. The emergency management scale was a self‐designed scale developed from literature on “emergency management,” which included 10 true‐or‐false questions; the higher the score represented, the better knowledge of emergency management. Content validity index (CVI) was used to evaluate the validity of the design of the questionnaire. The content was assessed by correctness, clarity, and readability. The score ranged from 1 to 4. The CVI of the scale was 0.94. The CVI was examined and analyzed by five experts, including three ICU nurses, one clinical nursing expert, two palliative care nurses, and one social worker (all with more than 15 years of experience).

Anxiety scale: The anxiety scale used here was derived from the State‐Trait Anxiety Inventory proposed by Zsido et al. [20]. The scale consisted of two dimensions with a total of 20 items. Four levels of answers were included, i.e., “not at all,” “a little,” “quite a bit,” and “very much,” which corresponds to 1–4 points, respectively. Positive and negative scaling was used, with items 1, 2, 5, 8, 10, 11, 15, 16, 19, and 20 in negative scoring. The score ranged from 20 to 80 points, and the lower score represented the mildest anxiety, i.e., 20–39 represented mild anxiety, 40–59 represented moderate anxiety, and 60–80 represented severe anxiety. Cronbach’s alpha for the situational Anxiety Scale was 0.83–0.92.

Mental Health Scale: The scale was developed based on the literature on mental health. It included 10 items; each scored from 1 to 5. The higher the score, the more stable the mental health. The Mental Health Scale was developed based on literature review, as no validated scale specifically designed for family members of terminally ill ICU patients in the Taiwanese cultural context was available at the time of study design. The scale was reviewed by experts to ensure content validity. Given its developmental nature, mental well‐being was positioned as a secondary outcome in this study.

### 2.4. Ethics

This study was approved by the institutional review board in Zuoying Armed Forces General Hospital (KAFGHIRB 110‐024). Participants were recruited after the IRB approved this study at Zuoying Armed Forces General Hospital. The researchers explained the study’s aim, procedure, and steps to the patients’ families during the recruitment. The study was started after obtaining the oral agreement and signed informed consent from all participants. The participant was free to withdraw from this study at any time. This study followed the research ethics, protected the subjects’ privacy, was conducted anonymously, and never disclosed the basic information of the participants. The noninvasive description of SDM was carried out on the premise of the family member’s wishes. Only one family member per patient was enrolled in the study to ensure independence of observations. For ethical fairness, the researchers explained SDM to the experimental and control groups at the end of this study.

### 2.5. Data Analysis

Data were assessed for normality using the Shapiro–Wilk test. As several variables exhibited non‐normal distributions, nonparametric statistical methods were employed: Mann–Whitney *U* test for between‐group comparisons and Wilcoxon signed‐rank test for within‐group pre–post comparisons. All statistical analyses were performed using SPSS version 25.0. A *p* value < 0.05 was considered statistically significant. Additionally, a multivariable linear regression analysis was conducted to adjust for the influence of potential confounding variables on the primary outcomes.

## 3. Results

**Figure 1:**
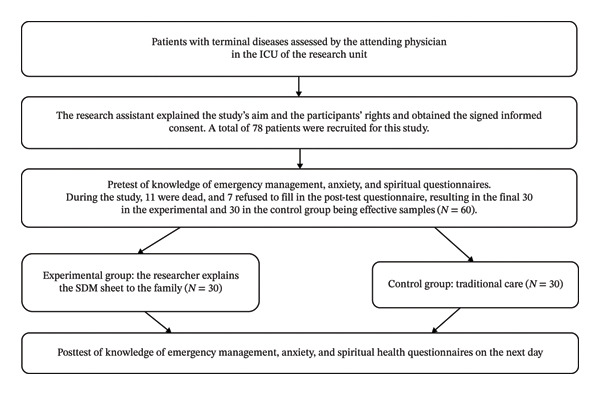
Flowchart of this study.

A total of 78 family members participated in this study. During the study period, 11 patients died (6 in the experimental group, 5 in the control group) and 7 family members declined to complete the post‐test questionnaire due to patient condition deterioration and family distress (*n* = 4), feeling overwhelmed by the decision‐making process (*n* = 2), or being unavailable for follow‐up (*n* = 1). This resulted in a final sample of 30 participants in each group (Figure 1).

Demographic characteristics are summarized in Supporting Information Table 1. The sample included 18 males (30%) and 42 females (70%), with ages ranging from 31 to 85 years (mean = 58.9, SD = 10.8). Chi‐squared and Fisher’s exact tests revealed no significant differences in demographic variables between groups, indicating high homogeneity. Analysis of prior experience with a relative’s death also showed no significant difference between the groups.

### 3.1. Baseline Characteristics of Emergency Management Knowledge, Anxiety, and Mental Health

At baseline, the mean emergency management knowledge score was 16.6 (SD = 3.49, range 10–20) out of a maximum of 20 points (Supporting Information Table 2). The mean anxiety score was 45.57 (SD = 13.37, range 22–75) out of 80 points (Supporting Information Table 3), indicating moderate anxiety levels. The mean mental health score was 32.98 (SD = 9.49, range 12–50) out of 100 points (Supporting Information Table 4).

Pretest comparisons using the Mann–Whitney *U* test (Table 1) revealed no significant differences between groups in emergency management knowledge or anxiety scores; however, an important difference was found in mental health scores (*p* = 0.01), with the experimental group scoring higher than the control group.

**Table 1:** Comparison of emergency management knowledge, anxiety, and mental health scores between experimental and control groups.

Outcome	Group	Pretest mean (SD) cc	Post‐test mean (SD)	Within‐group *p* value	Between‐group pretest *p* value	Between‐group post‐test *p* value
Emergency Management Knowledge	Experimental (*n* = 30)	16.87 (3.45)	19.33 (1.49)	> 0.05	0.385	> 0.05
	Control (*n* = 30)	16.33 (3.58)	17.17 (2.60)	> 0.05		

Anxiety	Experimental (*n* = 30)	45.40 (16.29)	47.13 (4.77)	> 0.05	0.842	0.284
	Control (*n* = 30)	45.73 (9.90)	43.63 (6.56)	> 0.05		

Mental Health	Experimental (*n* = 30)	36.80 (9.39)	34.77 (7.00)	> 0.05	0.01^∗^	> 0.05
	Control (*n* = 30)	29.17 (7.40)	26.43 (8.05)	> 0.05		

*Note:* Within‐group comparisons used Wilcoxon signed‐rank test; between‐group comparisons used the Mann–Whitney *U* test.

^∗^ *p* < 0.05.

### 3.2. Effect of SDM Intervention on Emergency Management Knowledge

Table 1 shows that emergency management knowledge scores increased in both groups from pretest to post‐test. In the experimental group, scores increased from 16.87 (SD = 3.45) to 19.33 (SD = 1.49). In the control group, scores increased from 16.33 (SD = 3.58) to 17.17 (SD = 2.60). Although the experimental group showed greater improvement, neither within‐group nor between‐group differences reached statistical significance (*p* > 0.05).

### 3.3. Effect of SDM Intervention on Anxiety

Table 1 shows that anxiety scores increased in the experimental group from 45.40 (SD = 16.29) to 47.13 (SD = 4.77), while they decreased slightly in the control group from 45.73 (SD = 9.90) to 43.63 (SD = 6.56). However, neither within‐group nor between‐group differences were statistically significant (*p* > 0.05).

### 3.4. Effect of SDM Intervention on Mental Health

Table 1 shows that mental health scores declined in both groups. In the experimental group, scores decreased from 36.80 (SD = 9.39) to 34.77 (SD = 7.00). In the control group, scores decreased from 29.17 (SD = 7.40) to 26.43 (SD = 8.05). Despite the baseline difference between groups (*p* = 0.01), neither within‐group nor between‐group post‐test differences reached statistical significance (*p* > 0.05).

## 4. Discussion

### 4.1. Current Status of Emergency Management Knowledge in Family Members of Terminally Ill Patients in ICU

The results of this study showed that the average emergency management knowledge score was 16.6 out of 20 points (83%), indicating a moderate to upper‐middle level of understanding. This differs from studies by Aaberg et al. [21] and Baldi et al. [22], where only 28% of medical students could correctly recognize normal breathing for basic life support. These discrepancies may reflect the growing emphasis on DNR education in clinical practice [22, 23]. A 2021 Uganda study showed that first‐aid training increased emergency management knowledge by 0.74 points, demonstrating the effectiveness of such interventions [24].

Item analysis revealed essential patterns. The highest correct‐response rates were for Q10 (post‐DNR patients still receive appropriate treatment, 77%), Q1 (CPR components, 72%), and Q9 (DNR reversibility, 71%). The lowest scores were for Q7 (post‐DNR treatment options, 53%), Q8 (palliative care definition, 57%), and Q3 (CPR complications, 59%). The low score on Q7 indicates clinicians should more clearly explain DNR choices to ensure families understand their decision‐making rights [25]. The misconception that palliative care means “giving up” underscores the need to reframe it as active, comfort‐oriented treatment [26]. As Holt et al. [27] noted, some families only consent to DNR after experiencing the burdens of aggressive interventions. These findings suggest that while family members have a moderate understanding, timely and tailored explanations of DNR and palliative care are essential for informed decision‐making.

### 4.2. Current Status of Anxiety in Family Members of Terminally Ill Patients in ICU

ICU admission of critically ill patients represents significant stress for families, who may experience anxiety, anger, depression, helplessness, and sleep disorders [28]. In this study, anxiety scores ranged from 22 to 75 (mean = 45.57), indicating moderate anxiety: 33.3% experienced mild anxiety, 48.3% moderate anxiety, and 18.4% severe anxiety. The COVID‐19 pandemic has intensified these issues through restricted hospital visits and reliance on video calls [29].

Studies consistently show that ICU family members experience significant anxiety, depression, and PTSD [30]. Research using the Hospital Anxiety and Depression Scale found average anxiety scores of 16.73, indicating clinically significant anxiety. This aligns with findings that clinical care often focuses on patients while overlooking the emotional needs of family members. There is a negative correlation between family satisfaction with ICU care and anxiety levels, with higher social support linked to lower anxiety [31]. The ICU’s closed environment, with its complex medical equipment, can cause family panic at any disturbance [32]. COVID‐19 further limited family access, allowing updates only through brief meetings or phone calls, amplifying stress.

### 4.3. Current Status of Mental Health in Family Members of Terminally Ill Patients in ICU

The average mental health score was 32.98 out of 50 (approximately 66%), indicating lower‐middle psychological well‐being. This contrasts with other populations who generally exhibit better mental health [28]. The COVID‐19 pandemic has exacerbated psychological distress due to increased stress and uncertainty [33].

Hope and social support are crucial protective factors helping families cope with ICU‐related stress [34]. Family involvement in care and effective communication with healthcare providers reduce psychological burden [35]. However, poor communication and caregiving stress can diminish hope and worsen mental health [34]. Virtual visiting during COVID‐19 reduced distress, but many still reported severe anxiety and depression [33]. Early screening for psychological distress and enhanced family‐centered care are essential [36]. The disclosure of mental illness and associated stigma can influence mental health, suggesting the need for tailored guidelines [37]. Overall, detailed and empathetic explanations by medical staff can support family members through this challenging period.

### 4.4. The Effect of SDM on Emergency Management Knowledge in Family Members of Terminally Ill Patients in ICU

SDM in the ICU has a significant impact on family knowledge. SDM involves cooperative information exchange between medical staff and families to make treatment decisions, emphasizing patient autonomy and family involvement [38, 39]. Analysis of the experimental group revealed that SDM intervention improved correct response rates for the lowest‐scoring items: Q7 (post‐DNR treatment options) increased by 30%, Q8 (palliative care definition) by 26.7%, and Q3 (CPR complications) by 33.3%. These substantial improvements suggest SDM effectively enhances emergency management knowledge. However, challenges remain, including uncertainty about long‐term outcomes and pressure on ICU physicians [40]. Ethnographic studies show that while clinicians present medical choices as a “buffet,” they recognize the complexity and emotional weight, guiding families through the process [41]. ICU nurses play a pivotal role as liaisons between physicians and families, ensuring patients’ values are integrated into decision‐making [40]. Substituted relational autonomy, where family members represent patient preferences, is crucial for respectful and ethical SDM [42]. The psychological impact on families underscores the need for high‐quality family‐centered care [43]. Overall, SDM enhances knowledge by providing information, emotional support, and validation, ultimately leading to more informed decisions.

### 4.5. Effect of SDM on Anxiety in Family Members of Terminally Ill Patients in the ICU

The study’s findings on SDM’s impact on anxiety reveal a paradoxical pattern. Despite the hypothesis that SDM would reduce anxiety, the experimental group’s anxiety increased from 45.40 to 47.13, while the control group’s anxiety decreased from 45.73 to 43.63, though differences were not statistically significant. This aligns with studies indicating that SDM can sometimes increase anxiety as families feel overwhelmed by decision‐making responsibility [44, 45]. The emotional burden of end‐of‐life decision‐making can be substantial. Surrogates often experience significant decisional conflict and regret, particularly when inadequately prepared for the patient’s death [45]. Family members may feel unqualified to make medical decisions and prefer relying on healthcare professionals’ expertise [46]. In this study, family members often deferred to doctors, saying, “Just do whatever you say!” Some cried or trembled while signing DNR forms, indicating profound stress [44].

These findings suggest that while SDM aims to respect patient autonomy and involve families, it can inadvertently increase anxiety without adequate support and clear communication. Effective SDM requires balancing information provision without overwhelming families, as too much information can exacerbate anxiety [40]. Healthcare professionals, particularly nurses, must offer emotional support and help families navigate decision‐making [46]. Despite the increased anxiety in the experimental group, the study highlights the importance of refining SDM practices, including preparing families for end‐of‐life decisions, enhancing emotional preparedness, and tailoring SDM to meet specific needs [45, 47].

### 4.6. Effects of SDM on Mental Health in Family Members of Terminally Ill Patients in ICU

Mental health scores decreased in both experimental (36.80–34.77) and control groups (29.17–26.43), though not significantly, suggesting that SDM may not have the anticipated positive impact [48]. This aligns with the literature, which indicates that families experience significant stress when making critical healthcare decisions [49]. Evaluating treatment options and life‐sustaining measures can be overwhelming, leaving little capacity for emotional self‐care [48, 50]. Involvement in SDM can lead to dissatisfaction if families feel inadequately supported. Neuro‐ICU research shows that fewer family meetings correlate with higher dissatisfaction, exacerbating stress and anxiety [2]. Comprehensive support systems, including frequent meetings and better communication about outcomes, are crucial [40]. While SDM aims to empower families, implementation often falls short due to time constraints, outcome uncertainty, and physician pressure [40]. These barriers limit inclusion and information sharing, critical for mental health and care satisfaction [2, 40].

Cultural context also plays a role. In some settings, familism culture and healthcare systems prioritize customer ideology over collaborative decision‐making, adding stress for families [48]. Future interventions should integrate emotional and spiritual support with SDM. Advanced care planning (ACP) interventions, especially family‐centered approaches, show promise in increasing familiarity with end‐of‐life options and improving discussions about life‐sustaining treatments [51]. Activities that enhance spiritual health can help individuals find meaning and purpose, potentially alleviating some of the emotional burdens associated with SDM [51]. Comprehensive support systems addressing both informational and emotional needs are essential for improving mental health outcomes.

## 5. Limitations

This study has several limitations. First, as a quasi‐experimental study, it is neither single‐ nor double‐blinded, and the nonrandomized design introduces potential for selection bias. Second, the Hawthorne effect may have influenced participant responses. Third, the Mental Health Scale was not a validated instrument, which is why we have positioned it as a secondary, exploratory outcome. Finally, we did not collect detailed data on certain potentially confounding variables, such as the specifics of the patient’s diagnosis trajectory or the family’s social support network.

## 6. Conclusions

This study found that a structured SDM intervention enhanced emergency management knowledge but did not alleviate anxiety or improve mental health among family members of terminally ill ICU patients. These findings suggest that SDM models must integrate targeted emotional and psychological support to be effective in high‐stress ICU environments. The research tools developed can be adapted by medical staff for clinical use. Future research should include larger, multicenter samples with random assignment, incorporate qualitative methods to understand family experiences better, and explore alternative SDM delivery formats, such as videos or interactive sessions, to improve accessibility and effectiveness, particularly for elderly participants.

## Author Contributions

Hui‐Ying Cheng, Shu Yuan Chao, and Hsin‐Hung Chen contributed to the study concept and design. Hui‐Ying Cheng was responsible for data acquisition. Shu Yuan Chao performed the data analysis and interpretation and provided statistical expertise. Hui‐Ying Cheng and Shu Yuan Chao drafted the initial manuscript. Both Shu Yuan Chao and Hsin‐Hung Chen conducted a critical revision of the manuscript for important intellectual content, which included final proofreading.

## Funding

This work was supported by the Zuoying Branch of Kaohsiung Armed Forces General Hospital (KAFGH‐ZY‐D‐111033) and the Zuoying Armed Forces General Hospital (ZYAFGH_D_115031).

## Conflicts of Interest

The authors declare no conflicts of interest.

## Supporting Information

Supporting Information and Results.

The supporting information presents detailed demographic characteristics of participants and pretest outcomes. These include participants’ educational level and baseline characteristics, the current status of emergency management knowledge, anxiety, and mental health among family members of terminally ill patients in the ICU, as well as the effects of the SDM intervention.

Supporting file 1: STROBE Statement—Checklist of items that should be included in reports of *cohort studies*.

Supporting Information Table 1. Basic demographic characteristics and homogeneity testing of the study participants.

Supporting Information Table 2. Pretest scores and item ranking of emergency management knowledge among family members of terminally ill ICU patients.

Supporting Information Table 3. Pretest anxiety scores and item ranking among family members of terminally ill ICU patients.

Supporting Information Table 4. Pretest mental health scores and item ranking among family members of terminally ill ICU patients.

## Supporting information


**Supporting Information** Additional supporting information can be found online in the Supporting Information section.

## Data Availability

The data that support the findings of this study are available on request from the corresponding author. The data are not publicly available due to privacy or ethical restrictions.
