# Supplementary material for: The Effect of Shared Decision‐Making on Emergency Management Knowledge, Anxiety, and Mental Health Among Family Members of Terminally Ill Patients in the ICU: A Quasiexperimental Study
Source: Nurs Res Pract. 2026 Mar 10;2026:8910437. doi: 10.1155/nrp/8910437 (PMC12973969; doi:10.1155/nrp/8910437)
Supplement: Supplementary file 1 — Supporting Information Additional supporting information can be found online in the Supporting Information section. [file NRP-2026-8910437-s001.zip › 251212-Supplementary Material Tables.docx]

**Supplementary Results**

The level of education in most of the participants was a university/college degree (n=25, 41.7%), followed by 12 at elementary school or below (20%) and 3 people (5%) at graduate school or above. Regarding the occupation status, most are retired, at home, and unemployed (n=37, 61.7%). Most were married (n=43, 71.7%). The Majority of participants had religious beliefs (n=50, 83.3%). The primary caregivers were children (n=34, 56.7%), followed by spouses and parents (n=7, 11.7%).

**The current situation of emergency management, anxiety, and mental health of family members of terminally ill patients in the ICU**

*Pre-test of family members of terminally ill patients in ICU in emergency management*

This study adopted a self-designed knowledge scale, with 10 true-or-false questions, 2 points for correct answers, and 1 point for wrong or unknown. The max score was 20 points. **Supplementary Material Table 2** shows that the scores of the subjects in the knowledge of emergency management ranged from 10-20 and averaged 16.6 (SD=3.49). Further analysis of the scores and ranking of each question in the scale showed that the items with the top three correct answer rates were "After signing the DNR, your family will still receive appropriate treatment" (77% accurate), "CPR refers to the treatment of patients who are dying or having vital signs by endotracheal intubation, extracorporeal heart compression, emergency drug injection, cardiac electric shock, artificial heart frequency modulation, artificial respiration, or other rescue measures" (72.1%), and "After signing the DNR, you can modify or retrieve it" (70.5% correct). The items with the lowest three correct answer rates were "After signing the DNR, you have treatment options, such as choosing to accept 'emergency drug injection' or refuse 'cardiac shock'" (52.5%), "Palliative care refers to the provision of palliative and supportive medical care, or do not perform CPR, to relieve the suffering of terminally ill patients" (57.4%), "CPR may result in rib fractures, chest electric shock burns, brain damage, deterioration of body functions, inability to regain consciousness, having to rely on life support machines, etc." (59.0%). Therefore, in the future, nursing care for the family members of patients can strengthen palliative care, emergency management options, and possible consequences after emergency management.

*Pre-test of family members of terminally ill patients in ICU in anxiety*

A total of 20 items were included in the anxiety scale with Likert four-point scoring (points 1 to 4 for each item, a total of 80 points). **Supplementary Material Table 3** showed that the score of anxiety ranged from 22 to 75, with an average of 45.57 (SD=13.37). Further analysis of the scores and raking of answers in each item showed that the top three items with the highest scoring rate are: "I regret" (M=3.18), "I feel unsatisfactory." (M=2.80), "I think too agitated and irritable." (M=2.67), "I feel oversensitive." (M=2.62), "I feel nervous." (M=2.57) and "I feel anxious." (M=2.57). The five items with the lowest scoring were "I feel joyful." (M=1.50), "I feel happy." (M=1.53), "I feel at ease." (M=1.73), " I am satisfied." (M=1.90), "I feel at ease." (M=1.92) and "I feel comfortable." (M=1.92).

*Pre-test of family members of terminally ill patients in ICU in mental health*

A total of 10 items were included in the mental health scale with Likert five-point scoring (points 1 to 5 for each item, total of 50 points). **Supplementary Material Table 4** showed that the mental health score ranged from 12 to 50, averaging 32.98 (SD=9.49). Further analysis of the scores and raking of answers in each item showed that the top five items with the highest scoring rate are "I feel trusted" (M=3.43), "I cannot blame others, I will do my best to deal with problems when I encounter problems, and I can accept the results regardless of it’s good or bad" (M =3.42), "I can still find a solution in a situation I don’t want to be in" (M=3.40); the three questions with the lowest scores are "I can rationally deal with the problems I encounter" (M= 3.23), "I accept the current unchangeable situation" (M=3.22), "I feel calm, peaceful and comforted” (M=2.98).

**Effect of sharing decision-making intervention on family members of terminally ill patients in the intensive care unit**

In examining the effectiveness of knowledge of emergency management, the number of correct answers (scores) among the 20 questions in the entire questionnaire was used as the dependent variable, and then the generalized estimating equation (GEE) was used to examine its effectiveness. In terms of knowledge of emergency management, anxiety, and mental health, the effect of the SDM intervention was compared via the Mann-Whitney and Wilcoxon tests.

**Supplementary Material Table 1. Basic information and homogeneity test of research subjects (N=60)**

| Basic information | Control group, N=30 (%) | Experimental group, N=30 (%) | Total, N=60 (%) | χ² | p |
| --- | --- | --- | --- | --- | --- |
| Gender |  |  |  | 1.270 | 0.260 |
| Male | 11 (36.7%) | 7 (23.3%) | 18 (30%) |  |  |
| Female | 19 (63.3%) | 23 (76.7%) | 42 (70%) |  |  |
| Religious belief |  |  |  | 4.000 | 0.406 |
| None | 3 (10%) | 7 (23.3%) | 10 (16.7%) |  |  |
| Christianity | 3 (10%) | 3 (10%) | 6 (10%) |  |  |
| Catholic | 2 (6.7%) | 0 (0%) | 2 (3.3%) |  |  |
| Buddhism | 19 (63.3%) | 16 (53.3%) | 35 (58.3%) |  |  |
| Taoism | 3 (10%) | 4 (13.3%) | 7 (11.7%) |  |  |
| Level of education |  |  |  | 5.440 | 0.369 |
| Illiterate | 2 (6.7%) | 2 (6.7%) | 4 (6.7%) |  |  |
| Elementary school | 9 (30%) | 3 (10%) | 12 (20%) |  |  |
| Junior high school | 4 (13.3%) | 2 (6.7%) | 6 (10%) |  |  |
| Senior and vocational high school | 4 (13.3%) | 6 (20%) | 10 (16.7%) |  |  |
| College/university | 10 (33.3%) | 15 (50%) | 25 (41.7%) |  |  |
| Graduate school or above | 1 (3.4%) | 2 (6.6%) | 3 (5%) |  |  |
| Occupation |  |  |  | 3.455 | 0.063 |
| Housekeeping, retired or unemployed | 22 (73.3%) | 15 (50%) | 37 (61.7%) |  |  |
| Student | 0 (0%) | 0 (0%) | 0 (0%) |  |  |
| Employed | 8 (26.7%) | 15 (50%) | 23 (38.3%) |  |  |
| Marital status |  |  |  | 1.309 | 0.727 |
| Single | 8 (26.7%) | 6 (20%) | 14 (23.3%) |  |  |
| Married/cohabitant | 21 (70%) | 22 (73.3%) | 43 (71.7%) |  |  |
| Divorced | 0 (0%) | 1 (3.3%) | 1 (1.67%) |  |  |
| Widowed | 1 (3.3%) | 1 (3.3%) | 2 (3.3%) |  |  |
| Relationship |  |  |  | 2.152 | 0.828 |
| Spouse | 4 (13.3%) | 3 (10%) | 7 (11.7%) |  |  |
| Child | 17 (56.7%) | 17 (56.7%) | 34 (56.7%) |  |  |
| Parent | 3 (10%) | 4 (13.3%) | 7 (11.7%) |  |  |
| Older brother/sister | 4 (13.3%) | 2 (6.7%) | 6 (10%) |  |  |
| Younger brother/sister | 2 (6.7%) | 3 (10%) | 5 (8.3%) |  |  |
| Other | 0 (0%) | 1 (3.3%) | 1 (1.7%) |  |  |
| Experiencing the death of a relative |  |  |  | 2.455 | 0.117 |
| Yes | 21 (70%) | 26 (86.7%) | 47 (78.3%) |  |  |
| No | 9 (30%) | 4 (13.3%) | 13 (21.7%) |  |  |
| Age (year) |  |  |  | z = -0.741 | 0.459 |
| Mean (SD) | 59.3 (11.713) | 58.5 (10.023) | 58.9 (10.815) |  |  |

All categorical variables were verified by Fisher Exact test; age was compared using Mann-Whitney U test.

**Supplementary Material Table 2**. **Scores and ranking of emergency management in the pre-test in family members of terminally ill patients** (N=60)

| Item | Correct | Probability of correct answer (%) | Ranking |
| --- | --- | --- | --- |
| 1. CPR refers to the treatment of patients who are dying or having vital signs by endotracheal intubation, extracorporeal heart compression, emergency drug injection, cardiac electric shock, artificial heart frequency modulation, artificial respiration, or other rescue measures | 44 | 72.1 | 2 |
| 1. The purpose of CPR is to maintain blood circulation by pressing the heart outside the body, so that oxygen can be supplied to vital organs. | 42 | 68.9 | 4 |
| 1. CPR may result in rib fractures, chest electric shock burns, brain damage, deterioration of body functions, inability to regain consciousness, having to rely on life support machines, etc | 36 | 59.0 | 8 |
| 1. If the heartbeat stops suddenly and no treatment is carried out, the brain will be damaged after 4 to 6 minutes of hypoxia; if there is no first aid measure in 10 minutes or more, it will cause brain damage that cannot be recovered. | 38 | 62.3 | 6 |
| 1. There is a domestic law that when a doctor diagnoses a patient as terminally ill and the treatment is ineffective, the patient has the right to decide "not to receive emergency management". | 37 | 60.7 | 7 |
| 1. Cardiac arrest can easily affect blood circulation throughout the body, resulting in severe hypoxia, tissue necrosis, and acidosis. Even if the endotracheal intubation delivers oxygen immediately, as well as external heart massage and electric shock, the heart function can be temporarily restored, but the blood circulation in the brain may still be affected, and the prognosis will still be poor or even death. | 42 | 68.9 | 4 |
| 1. After signing the DNR, you have treatment options, such as choosing to accept 'emergency drug injection' or refuse 'cardiac shock | 32 | 52.5 | 10 |
| 1. Palliative care refers to the provision of palliative and supportive medical care, or do not perform CPR, in order to relieve the suffering of terminally ill patients | 35 | 57.4 | 9 |
| 1. After signing the DNR, you can modify or retrieve it | 43 | 70.5 | 3 |
| 1. After signing the DNR, your family will still receive appropriate treatment | 47 | 77.0 | 1 |

**Supplementary Material Table 3. Scores and ranking of anxiety in the pre-test in family members of terminally ill patients** (N=60)

| Item | Min | Max | Mean | SD | Ranking |
| --- | --- | --- | --- | --- | --- |
| 1. I feel calm. | 1 | 4 | 2.18 | 0.930 | 12 |
| 2. I feel safe. | 1 | 4 | 2.47 | 0.982 | 8 |
| 3. I am nervous. | 1 | 4 | 2.57 | 0.945 | 5 |
| 4. I regret. | 1 | 4 | 3.18 | 0.596 | 1 |
| 5. I feel at ease. | 1 | 4 | 1.92 | 0.907 | 15 |
| 6. I feel unsatisfactory. | 1 | 4 | 2.80 | 1.038 | 2 |
| 7. I now worry about possible mishaps. | 1 | 4 | 2.47 | 1.112 | 8 |
| 8. I feel relaxed. | 1 | 4 | 1.73 | 0.861 | 18 |
| 9. I feel anxious. | 1 | 4 | 2.57 | 1.031 | 5 |
| 10. I feel comfortable. | 1 | 4 | 1.92 | 0.926 | 15 |
| 11. I feel confident. | 1 | 4 | 2.13 | 0.911 | 13 |
| 12. I feel distressed. | 1 | 4 | 2.47 | 1.016 | 8 |
| 13. I feel oversensitive. | 1 | 4 | 2.62 | 1.106 | 4 |
| 14. I feel nervous. | 1 | 4 | 2.57 | 1.110 | 5 |
| 15. I feel good. | 1 | 4 | 1.98 | 1.049 | 14 |
| 16. I feel satisfied. | 1 | 4 | 1.90 | 1.037 | 17 |
| 17. I am worried. | 1 | 4 | 2.40 | 0.942 | 11 |
| 18. I feel too agitated and irritable. | 1 | 4 | 2.67 | 1.084 | 3 |
| 19. I feel happy. | 1 | 4 | 1.53 | 0.892 | 19 |
| 20. I feel joyful. | 1 | 4 | 1.50 | 0.893 | 20 |

**Supplementary Material Table 4. Scores and rankings of the mental health of the patient's family members** (N=60)

| Item | Minimum | Maximum | Mean | SD | Ranking |
| --- | --- | --- | --- | --- | --- |
| 1. I feel calm, peaceful and comforted. | 1 | 5 | 2.98 | 1.308 | 10 |
| 1. I can rationally deal with the problems I encounter | 1 | 5 | 3.23 | 1.240 | 9 |
| 1. I accept the current unchangeable situation. | 1 | 5 | 3.22 | 1.274 | 8 |
| 1. I can still find a solution in a situation I don’t want to be in. | 1 | 5 | 3.40 | 1.012 | 3 |
| 1. I can live in the present and will try to let go of the past and anxiety about the future. | 1 | 5 | 3.38 | 0.904 | 4 |
| 1. I cannot blame others; I will do my best to deal with problems when I encounter them and accept the results regardless of whether they are good or bad. | 1 | 5 | 3.42 | 1.094 | 2 |
| 1. I can learn to let go and feel at peace and belonging. | 1 | 5 | 3.35 | 1.055 | 5 |
| 1. I feel trusted. | 1 | 5 | 3.43 | 1.079 | 1 |
| 1. I can give unconditionally, am not afraid of difficulties, and accept what I cannot change. | 1 | 5 | 3.28 | 1.091 | 7 |
| 1. I can calm myself down by my inner strength. | 1 | 5 | 3.32 | 1.097 | 6 |
